# Supplementary material for: Model confidence sets and forecast combination: an application to age-specific mortality
Source: Genus. 2018 Nov 21;74(1):19. doi: 10.1186/s41118-018-0043-9 (PMC6276067; doi:10.1186/s41118-018-0043-9)
Supplement: Supplementary file 2 — Detailed point and interval forecast results. While Table 6 presents a summary of the point and interval forecast accuracies, we present the detailed forecast results for ten years in the forecasting period. Calculation for single-premium fixed-term immediate annuity. The forecasted mortality rate is an essential input for determining temporary annuity prices for various maturities and starting ages of the annuitant. (PDF 149 kb) [file 41118_2018_43_MOESM2_ESM.pdf]

Supplement to "Model confidence sets and forecast combination: An application to mortality forecasting"

S1. Geographical locations of the 47 prefectures

We plot geographic locations (from North to South) of the 47 prefectures within eight regions of Japan in Figure 1. Also, we present the names of the prefectures within each of the eight regions of Japan in Table 1.

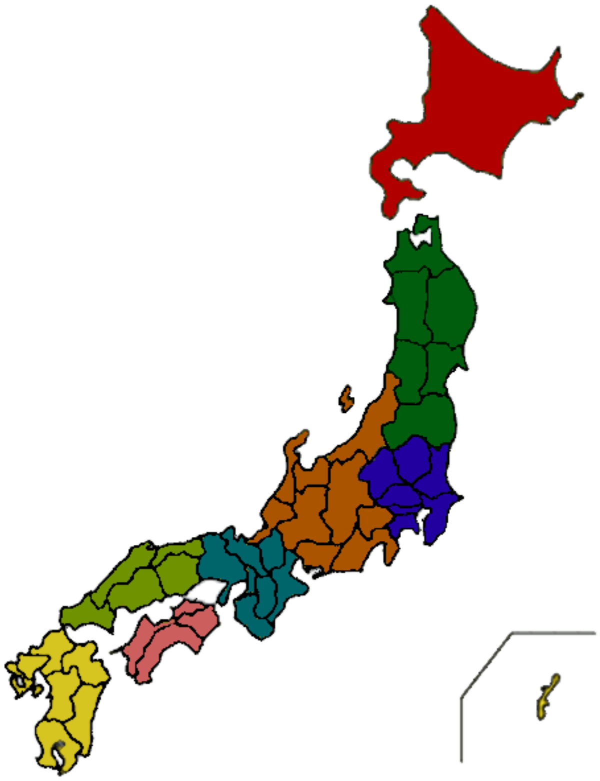

Figure 1: *Geography locations of the 47 prefectures within eight regions of Japan.*
Source: https://en.wikipedia.org/wiki/Prefectures_of_Japan

Table 1: *Name of the prefectures within each region of Japan. The regions and the prefectures are ordered geographically from North to South.*

Prefecture within each region							
Hokkaidō	Tōhoku	Kantō	Chūbu	Kansai	Chōgoku	Shikoku	Kyūshū
Hokkaidō	Aomori	Ibaraki	Niigata	Mie	Tottori	Tokushima	Fukuoka
	Iwate	Tochigi	Toyama	Shiga	Shimane	Kagawa	Saga
	Miyagi	Gunma	Ishikawa	Kyōto	Okayama	Ehime	Nagasaki
	Akita	Saitama	Fukui	Ōsaka	Hiroshima	Kōchi	Kumamoto
	Yamagata	Chiba	Yamanashi	Hyōgo	Yamaguchi		Ōita
	Fukushima	Tōkyō	Nagano	Nara			Miyazaki
		Kanagawa	Gifu	Wakayama			Kagoshima
			Shizuoka				Okinawa
			Aichi				

S2. Detailed point and interval forecast results

While a summary of the point and interval forecast accuracies is given in Table 6, we report the detailed point and interval forecast errors for the ten years in the forecasting period in Tables 2, 3 and 4, 5.

Table 2: *Point forecast accuracy among the 17 models and two model-averaged methods in Japan for ages between 60 and 100+. Forecast errors have been multiplied by 100. The smallest overall errors are highlighted in bold.*

Series	Method	2006	2007	2008	2009	2010	2011	2012	2013	2014	2015	Mean
Female	1	0.41	0.37	0.61	0.25	0.86	0.86	0.73	0.60	0.25	0.43	0.54
	2	1.45	1.59	2.24	2.75	3.15	4.90	5.22	6.92	6.88	6.86	4.20
	3	0.99	1.08	1.49	1.08	1.85	1.73	1.47	1.42	1.02	1.25	1.34
	4	1.72	1.85	2.11	1.67	2.33	2.28	2.22	1.96	1.72	1.97	1.98
	5	0.98	1.04	1.37	0.92	1.60	1.51	1.39	1.14	0.95	1.09	1.20
	6	1.93	1.97	2.28	1.81	2.42	2.29	2.20	2.00	1.69	1.93	2.05
	7	0.81	0.82	1.02	0.71	1.14	1.09	0.98	0.73	0.78	0.66	0.87
	8	0.19	0.21	0.54	0.11	0.75	0.47	0.35	0.12	0.35	0.21	**0.33**
	9	0.48	0.43	0.70	0.38	0.97	1.02	0.88	0.80	0.46	0.62	0.67
	10	0.48	0.43	0.71	0.39	0.99	1.05	0.92	0.85	0.49	0.65	0.69
	11	0.47	0.42	0.69	0.38	0.98	1.04	0.90	0.85	0.50	0.65	0.69
	12	0.46	0.41	0.68	0.38	0.97	1.05	0.90	0.89	0.53	0.67	0.69
	13	0.69	0.67	0.89	0.59	0.88	0.98	0.81	0.48	0.65	0.45	0.71
	14	0.68	0.67	0.89	0.59	0.99	1.02	0.83	0.48	0.65	0.45	0.72
	15	0.54	0.54	0.67	0.46	0.82	0.88	0.67	0.45	0.67	0.45	0.61
	16	0.64	0.68	0.89	0.59	0.97	1.16	1.08	0.88	0.74	0.68	0.83
	17	0.72	0.63	0.80	0.57	1.01	1.16	1.11	0.80	0.68	0.62	0.81
	18	0.41	0.37	0.61	0.25	0.86	0.86	0.73	0.60	0.25	0.43	0.54
	19	0.41	0.37	0.61	0.25	0.86	0.86	0.73	0.60	0.25	0.43	0.54
Male	1	0.42	0.34	1.07	0.44	1.26	1.14	1.06	0.53	0.38	0.44	0.71
	2	0.68	0.91	0.58	1.51	1.05	1.76	2.97	4.61	4.87	3.70	2.27
	3	1.05	1.31	2.24	1.47	2.75	2.56	2.44	1.95	1.66	1.92	1.93
	4	2.18	2.45	3.36	2.37	3.56	3.30	3.43	2.83	2.64	3.08	2.92
	5	1.13	1.36	2.29	1.32	2.54	2.21	2.23	1.60	1.36	1.80	1.78
	6	2.33	2.50	3.47	2.30	3.54	3.10	3.09	2.36	2.25	2.58	2.75
	7	1.12	1.31	2.21	1.18	2.33	1.98	1.98	1.32	1.06	1.49	1.60
	8	0.90	0.73	0.63	0.93	0.75	0.48	0.25	0.75	0.64	0.39	**0.65**
	9	0.55	0.54	1.35	0.56	1.61	1.51	1.49	0.94	0.57	0.78	0.99
	10	0.55	0.53	1.35	0.56	1.62	1.54	1.52	0.97	0.60	0.80	1.00
	11	0.57	0.57	1.38	0.58	1.64	1.56	1.52	0.99	0.61	0.81	1.02
	12	0.54	0.55	1.37	0.56	1.62	1.52	1.49	0.95	0.60	0.80	1.00
	13	0.29	0.43	1.28	0.61	1.17	1.10	0.74	0.49	0.49	0.63	0.72
	14	0.59	0.63	1.49	0.57	1.20	1.03	0.71	0.48	0.48	0.63	0.78
	15	0.31	0.38	1.30	0.58	1.21	1.07	0.80	0.48	0.45	0.62	0.72
	16	0.53	0.50	1.35	0.64	1.41	1.46	1.38	0.79	0.51	0.74	0.93
	17	0.52	0.43	1.33	0.58	1.25	0.89	0.62	0.64	0.69	0.65	0.76
	18	0.42	0.34	1.07	0.44	1.26	1.14	1.06	0.53	0.38	0.44	0.71
	19	0.42	0.34	1.07	0.44	1.26	1.14	1.06	0.53	0.38	0.44	0.71

Table 3: *Point forecast accuracy among the 17 models and two model-averaged methods, averaged across the 47 sub-national populations for ages between 60 and 100+. Forecast errors have been multiplied by 100. The smallest errors are highlighted in bold.*

Series	Method	2006	2007	2008	2009	2010	2011	2012	2013	2014	2015	Mean
Female	1	1.16	1.12	1.21	1.05	1.30	1.30	1.26	1.04	0.84	0.82	1.11
	2	2.00	2.27	2.61	2.88	3.79	4.41	7.52	8.01	7.97	20.98	6.24
	3	1.40	1.46	1.72	1.38	2.03	1.96	1.86	1.60	1.25	1.40	1.61
	4	2.03	2.10	2.30	1.87	2.49	2.47	2.42	2.12	1.88	2.08	2.17
	5	1.44	1.44	1.65	1.31	1.84	1.77	1.69	1.40	1.23	1.31	1.51
	6	2.23	2.24	2.47	2.02	2.62	2.50	2.43	2.19	1.89	2.09	2.27
	7	1.33	1.29	1.41	1.19	1.46	1.41	1.36	1.12	1.13	1.01	1.27
	8	1.09	1.07	1.15	1.02	1.24	1.07	1.01	0.87	0.89	0.79	**1.02**
	9	1.33	1.23	1.32	1.20	1.42	1.46	1.40	1.23	1.01	0.99	1.26
	10	1.33	1.23	1.32	1.20	1.42	1.48	1.42	1.26	1.02	1.01	1.27
	11	1.32	1.23	1.31	1.20	1.41	1.47	1.45	1.26	1.02	1.01	1.27
	12	1.32	1.22	1.31	1.21	1.41	1.47	1.48	1.29	1.03	1.03	1.28
	13	1.19	1.18	1.27	1.10	1.33	1.46	1.45	1.18	1.04	0.90	1.21
	14	1.21	1.20	1.25	1.09	1.31	1.46	1.46	1.20	1.04	0.91	1.21
	15	1.18	1.19	1.24	1.12	1.32	1.43	1.48	1.16	1.05	0.92	1.21
	16	1.21	1.21	1.26	1.14	1.34	1.45	1.51	1.25	1.09	1.01	1.25
	17	1.22	1.21	1.24	1.16	1.30	1.44	1.44	1.22	1.07	0.96	1.23
	18	1.15	1.15	1.24	1.09	1.34	1.39	1.54	1.31	1.20	0.96	1.24
	19	1.16	1.16	1.26	1.07	1.35	1.38	1.55	1.27	1.04	0.91	1.22
Male	1	2.80	2.82	2.65	2.74	2.74	2.82	2.59	2.20	2.14	2.03	2.55
	2	3.46	3.50	3.23	3.61	3.50	3.87	4.23	4.71	5.58	4.48	4.02
	3	2.83	2.89	3.25	2.96	3.72	3.73	3.54	3.08	2.61	2.72	3.13
	4	3.31	3.50	3.94	3.40	4.24	4.27	4.15	3.80	3.37	3.65	3.76
	5	2.75	2.83	3.15	2.75	3.46	3.43	3.21	2.89	2.48	2.63	2.96
	6	3.47	3.60	4.11	3.43	4.33	4.16	3.91	3.46	3.12	3.28	3.69
	7	2.76	2.78	3.10	2.68	3.28	3.27	3.01	2.75	2.32	2.42	2.84
	8	2.90	2.68	2.51	2.69	2.65	2.58	2.32	2.30	2.12	1.96	2.47
	9	3.73	3.81	4.08	3.79	4.38	4.19	3.88	3.61	3.12	3.23	3.78
	10	3.69	3.75	4.04	3.76	4.34	4.17	3.88	3.61	3.12	3.23	3.76
	11	3.76	3.82	4.11	3.82	4.41	4.25	3.98	3.67	3.17	3.27	3.83
	12	3.31	3.43	3.75	3.45	4.01	3.83	3.65	3.34	2.90	3.02	3.47
	13	2.54	2.51	2.56	2.49	2.76	2.99	2.72	2.46	2.11	1.98	2.51
	14	2.54	2.51	2.55	2.49	2.76	2.98	2.71	2.46	2.12	1.99	2.51
	15	2.54	2.48	2.51	2.46	2.71	2.88	2.62	2.40	2.07	1.98	2.47
	16	2.55	2.48	2.51	2.47	2.67	2.89	2.64	2.43	2.07	1.94	2.47
	17	2.66	2.57	2.48	2.50	2.58	2.73	2.38	2.29	2.19	2.03	**2.44**
	18	2.64	2.59	2.57	2.48	2.82	2.89	2.65	2.37	2.06	2.00	2.51
	19	2.65	2.58	2.57	2.50	2.80	2.87	2.65	2.37	2.05	1.97	2.50

Table 4: *Interval forecast accuracy among the 17 models and two model-averaged methods in Japan for ages between 60 and 100+. Forecast errors have been multiplied by 100. The smallest overall errors are highlighted in bold.*

Series	Method	2006	2007	2008	2009	2010	2011	2012	2013	2014	2015	Mean
Female	1	1.04	0.90	1.90	0.67	3.50	3.38	2.53	2.17	0.67	1.39	1.81
	2	3.54	4.36	6.62	5.84	10.45	8.72	6.33	8.05	5.86	11.44	7.12
	3	2.84	3.75	5.36	3.77	7.67	6.73	5.46	5.15	3.51	4.86	4.91
	4	5.67	6.51	7.82	5.71	9.35	8.77	8.21	7.07	5.90	7.37	7.24
	5	2.42	3.21	4.67	2.23	5.94	5.32	4.49	3.14	2.33	3.35	3.71
	6	5.09	6.10	6.95	4.88	8.27	7.24	6.60	5.47	3.56	5.35	5.95
	7	1.89	1.89	2.14	1.78	2.16	2.27	2.20	1.77	1.63	1.60	1.93
	8	0.76	0.80	0.79	0.79	1.48	0.94	0.79	0.74	0.75	0.76	**0.86**
	9	1.14	1.04	1.96	1.00	3.68	3.52	2.84	2.62	1.13	2.04	2.10
	10	1.14	1.05	2.01	1.02	3.82	3.81	3.06	2.90	1.32	2.27	2.24
	11	1.13	1.02	1.94	1.01	3.75	3.72	2.95	2.93	1.33	2.24	2.20
	12	1.11	0.97	1.85	1.00	3.68	3.75	2.91	3.11	1.45	2.33	2.22
	13	1.19	1.16	1.50	1.05	1.85	2.45	1.40	0.79	1.01	0.68	1.31
	14	1.17	1.15	1.49	1.05	2.14	2.60	1.46	0.77	0.99	0.68	1.35
	15	1.32	1.30	1.27	1.28	1.27	1.41	1.37	1.36	1.33	1.28	1.32
	16	1.17	1.21	1.41	1.00	1.89	2.46	1.84	1.15	1.04	1.12	1.43
	17	1.51	1.31	1.60	1.26	2.45	2.57	2.56	1.72	1.58	1.44	1.80
	18	0.79	0.77	0.81	0.83	1.55	0.91	0.79	0.75	0.76	0.69	0.87
	19	0.79	0.77	0.81	0.83	1.55	0.91	0.79	0.75	0.76	0.69	0.87
Male	1	1.26	0.96	3.68	1.44	5.74	5.19	4.67	2.05	1.30	1.63	2.79
	2	2.13	2.25	1.88	5.47	2.97	5.04	10.20	17.70	18.76	13.33	7.97
	3	3.89	5.32	9.57	5.88	12.29	11.35	10.75	8.21	6.76	7.63	8.17
	4	8.27	10.25	14.00	9.17	15.59	14.13	14.53	11.57	11.05	13.28	12.18
	5	3.05	5.12	8.87	4.38	10.70	8.60	8.59	4.99	4.00	5.92	6.42
	6	7.14	9.14	13.17	7.45	14.15	11.27	11.20	7.53	7.30	8.84	9.72
	7	2.52	2.87	5.34	2.82	6.74	4.99	4.95	2.65	2.15	3.10	3.81
	8	1.52	1.47	1.19	1.81	1.68	1.20	1.18	1.31	1.16	1.16	1.37
	9	1.52	1.52	4.56	1.70	6.79	6.27	5.97	3.49	2.06	2.93	3.68
	10	1.53	1.51	4.59	1.73	6.92	6.55	6.19	3.72	2.20	3.06	3.80
	11	1.56	1.67	4.82	1.78	7.09	6.71	6.25	3.83	2.28	3.11	3.91
	12	1.46	1.50	4.48	1.66	6.69	6.16	5.84	3.46	2.09	2.96	3.63
	13	1.18	1.12	2.01	1.36	2.33	2.19	1.29	1.18	1.16	1.14	1.50
	14	1.17	1.08	2.50	1.25	2.26	1.85	1.28	1.18	1.14	1.14	1.49
	15	1.96	1.90	2.30	1.89	2.04	1.93	2.00	1.99	1.92	1.87	1.98
	16	1.77	1.67	1.61	1.62	1.77	2.23	1.84	1.77	1.87	1.75	1.79
	17	1.51	1.30	2.38	1.51	2.41	1.71	1.61	1.76	1.87	1.87	1.79
	18	1.54	1.54	1.24	1.80	1.58	1.26	1.20	1.30	1.10	1.05	**1.36**
	19	1.54	1.54	1.24	1.80	1.58	1.26	1.20	1.30	1.10	1.05	**1.36**

Table 5: *Interval forecast accuracy among the 17 models and two model-averaged methods, averaged across the 47 sub-national populations for ages between 60 and 100+. Forecast errors have been multiplied by 100. The smallest overall errors are highlighted in bold.*

Series	Method	2006	2007	2008	2009	2010	2011	2012	2013	2014	2015	Mean
Female	1	3.86	3.67	4.01	3.65	4.73	4.96	4.65	3.55	2.86	2.87	3.88
	2	4.80	5.07	6.26	5.94	8.00	7.96	40.38	42.10	32.27	464.22	61.70
	3	4.30	4.62	5.69	4.40	7.33	7.20	6.68	5.23	3.94	4.70	5.41
	4	6.18	6.55	7.50	5.68	8.74	8.68	8.11	6.75	5.72	6.97	7.09
	5	3.99	4.14	5.05	3.60	6.12	5.87	5.26	3.90	3.29	3.83	4.50
	6	5.48	5.74	6.56	4.85	7.58	7.02	6.49	5.23	4.25	5.19	5.84
	7	3.04	2.87	3.08	2.77	3.15	3.38	3.21	2.57	2.53	2.28	2.89
	8	2.46	2.36	2.59	2.43	2.88	2.80	2.60	1.99	2.02	1.84	**2.40**
	9	4.37	3.99	4.30	4.10	4.94	5.22	5.00	4.02	3.27	3.37	4.26
	10	4.41	4.01	4.34	4.14	4.99	5.38	5.15	4.17	3.36	3.47	4.34
	11	4.40	3.99	4.28	4.15	4.91	5.27	5.34	4.18	3.34	3.46	4.33
	12	4.30	3.88	4.20	4.11	4.81	5.21	5.43	4.26	3.35	3.50	4.30
	13	2.55	2.42	2.66	2.40	2.82	3.46	3.49	2.76	2.31	2.25	2.71
	14	2.58	2.41	2.64	2.39	2.82	3.42	3.49	2.72	2.31	2.25	2.70
	15	3.00	2.91	2.84	2.83	2.82	3.21	3.34	2.94	2.80	2.72	2.94
	16	3.46	3.32	3.48	3.15	3.53	4.08	3.87	3.18	2.96	2.94	3.40
	17	2.73	2.67	2.72	2.54	2.78	3.36	3.14	2.71	2.40	2.29	2.73
	18	2.61	2.49	2.60	2.40	2.69	2.91	3.10	2.52	2.24	2.15	2.57
	19	2.65	2.53	2.61	2.41	2.71	2.89	3.05	2.50	2.23	2.13	2.57
Male	1	10.28	10.15	9.84	10.27	10.40	10.94	9.97	8.04	8.07	7.68	9.56
	2	11.47	11.61	11.26	12.40	11.96	14.02	15.89	18.29	22.18	16.62	14.57
	3	10.11	10.39	12.15	10.44	14.18	14.22	13.16	10.55	9.11	9.65	11.40
	4	10.46	11.83	13.77	10.93	15.44	15.42	14.87	12.70	11.44	13.22	13.01
	5	8.34	9.10	10.46	8.40	11.98	11.62	10.47	8.55	7.35	8.23	9.45
	6	9.32	10.31	12.33	9.25	13.44	12.53	11.28	9.09	8.20	8.99	10.47
	7	7.80	7.72	8.67	7.44	9.49	9.44	8.62	7.23	6.09	6.57	7.91
	8	6.85	6.12	6.20	6.23	6.23	6.26	5.46	5.14	4.80	4.37	5.77
	9	13.05	12.81	13.53	12.75	14.64	14.59	13.55	11.76	10.54	10.80	12.80
	10	12.97	12.66	13.42	12.66	14.51	14.62	13.62	11.86	10.59	10.86	12.78
	11	13.21	12.94	13.73	12.90	14.90	14.98	14.02	12.05	10.76	10.98	13.05
	12	10.78	10.52	11.27	10.83	12.14	12.27	11.83	10.29	9.24	9.63	10.88
	13	6.14	5.78	5.80	5.68	6.00	6.66	6.38	5.57	5.35	5.28	5.86
	14	6.15	5.76	5.79	5.68	6.01	6.67	6.39	5.58	5.35	5.28	5.87
	15	5.23	5.02	5.02	4.91	5.25	5.62	5.41	4.94	4.48	4.32	**5.02**
	16	7.65	7.46	7.51	7.19	7.15	7.46	7.45	7.04	6.74	6.61	7.23
	17	5.92	5.66	5.54	5.47	5.74	6.05	5.37	5.20	5.02	4.94	5.49
	18	5.22	5.01	5.07	4.94	5.18	5.71	5.50	5.02	4.53	4.43	5.06
	19	5.24	5.03	5.07	4.94	5.19	5.67	5.50	5.02	4.54	4.46	5.07

S3. Application to single-premium fixed-term immediate annuity

An important use of mortality forecasts for the elderly (at ages approximately older than 60 years of age) is in the pension and insurance industries, whose profitability and financial strength depend on accurate mortality forecasts to appropriately hedge longevity risks. When a person retires, an optimal way of guaranteeing one individual's financial income in retirement is to purchase an annuity (as demonstrated by Yaari, 1965). An annuity is a contract offered by insurers guaranteeing a steady stream of payments for either a fixed term or the lifetime of the annuitants in exchange for an initial premium fee.

Lifetime immediate annuities, where rates are locked in for life, have been shown to deliver poor value for money (i.e., they may be expensive for the purchaser: see, e.g., Cannon and Tonks, 2008, Chapter 6). We consider fixed-term annuities, which have grown in popularity in some countries. These fixed-term annuities pay a pre-determined and guaranteed level of income which is higher than the level of income provided by a lifetime annuity for a similar premium. Fixed-term annuities offer an alternative to lifetime annuities and allow the purchaser the option of also buying a deferred annuity to start at a later date. By using the constraint that the last age of the fixed-term annuity is less than 100, we also avoid the problem of extrapolating the sets of mortality rates up to the very highest ages.

We apply the mortality forecasts to the calculation of a fixed-term annuity (see Dickson et al., 2009, p. 114), and we adopt a cohort approach to the calculation of survival probabilities. For a single cohort, the τ year survival probability of a person aged x currently at $t = 0$ (or year 2016) is determined by

$$_{\tau}p_x = \prod_{j=1}^{\tau} {}_1p_{x+j-1} = \prod_{j=1}^{\tau} \exp^{-\mathcal{Y}_{x+j-1,j-1}},$$

which is a random variable given that mortality rates for $j = 1, \dots, \tau$ are forecasts obtained by a model-averaging method based on the $T_{\text{max,M}}$ test. Here, we assume that the central mortality rates are constant throughout each one-year period.

The price of an annuity with maturity T year, written for an x-year-old with benefit \$1 per year and conditional on the path is given by

$$a_x^T(\mathcal{Y}_{1:\tau}^x) = \sum_{\tau=1}^{T} B(0,\tau)\mathrm{E}(\mathbb{1}_{T_x>\tau}|\mathcal{Y}_{1:\tau}^x) = \sum_{\tau=1}^{T} B(0,\tau)_{\tau}p_x(\mathcal{Y}_{1:\tau}^x),$$

where $B(0,\tau)$ is the τ-year bond price, $\mathcal{Y}_{1:\tau}^x$ is the first τ elements of $\mathcal{Y}_{1:T}^x$, and $_{\tau}p_x(\mathcal{Y}_{1:\tau})^x$ denotes the survival probability given a random $\mathcal{Y}_{1:\tau}^x$ (see also Fung et al., 2017). For the purpose of pricing, reserving and risk management, it is vital to produce an accurate forecast of the survival curve $_{\tau}p_x$ that best captures the mortality experience of a portfolio.

In Table 6, to provide an example of the annuity calculations, we present the model-averaged point forecasts of the annuity prices using the $T_{\text{max,M}}$ test for different ages and maturities for a female policyholder residing in Akita, where we have single years of observation from 1975 to 2015. We assume a constant interest rate at $\eta = 3\%$, and hence $B(0, \tau) = \exp^{-\eta\tau}$.

Table 6: *Estimates of annuity prices with different ages and maturities (T) for a female policyholder residing in prefecture Akita. These estimates are based on forecasting age-specific mortality rates from 2016 to 2056. We consider only contracts with maturity so that age + maturity ≤ 100. If age + maturity > 100, NA will be shown in the table.*

Age	$T = 5$	$T = 10$	$T = 15$	$T = 20$	$T = 25$	$T = 30$
60	4.53	8.34	11.52	14.13	16.19	17.65
65	4.51	8.28	11.37	13.80	15.54	16.56
70	4.49	8.17	11.07	13.14	14.36	14.87
75	4.43	7.92	10.41	11.87	12.49	NA
80	4.30	7.37	9.18	9.94	NA	NA
85	4.01	6.38	7.37	NA	NA	NA
90	3.53	5.01	NA	NA	NA	NA
95	2.89	NA	NA	NA	NA	NA

To quantify forecast uncertainty, we construct the model-averaged prediction interval of mortality rates, derive the survival probabilities and calculate the annuities for different ages and maturities in Table 7. Since the superior set of models included in the proposed model-averaging methods are different for producing point and interval forecasts of mortality rates, the point forecasts of the annuity price do not necessarily lie with the constructed pointwise prediction intervals.

Table 7: *95% pointwise prediction intervals of annuity prices with different ages and maturities (T) for female policyholder residing in prefecture Akita, for example. These estimates are based on forecast mortality rates for ages between 60 and 100+ from 2016 to 2056. We consider only contracts with maturity so that age + maturity ≤ 100. If age + maturity > 100, NA will be shown in the table.*

Age	$T = 5$	$T = 10$	$T = 15$	$T = 20$	$T = 25$	$T = 30$
60	(4.52, 4.53)	(8.32, 8.36)	(11.50, 11.57)	(14.11, 14.23)	(16.17, 16.38)	(17.68, 17.99)
65	(4.50, 4.52)	(8.26, 8.32)	(11.35, 11.47)	(13.79, 14.01)	(15.57, 15.92)	(16.68, 17.13)
70	(4.47, 4.51)	(8.15, 8.25)	(11.05, 11.27)	(13.18, 13.52)	(14.48, 14.99)	(15.13, 15.71)
75	(4.41, 4.48)	(7.90, 8.09)	(10.44, 10.80)	(12.01, 12.54)	(12.77, 13.43)	NA
80	(4.28, 4.40)	(7.39, 7.71)	(9.31, 9.85)	(10.23, 10.94)	NA	NA
85	(4.02, 4.21)	(6.49, 6.96)	(7.65, 8.34)	NA	NA	NA
90	(3.55, 3.86)	(5.21, 5.83)	NA	NA	NA	NA
95	(2.94, 3.36)	NA	NA	NA	NA	NA

References

Cannon, E. and Tonks, I. (2008), *Annuity Markets*, Oxford University Press, Oxford.

Dickson, D. C. M., Hardy, M. R. and Waters, H. R. (2009), *Actuarial Mathematics for Life Contingent Risks*, Cambridge University Press, Cambridge.

Fung, M. C., Peters, G. W. and Shevchenko, P. V. (2017), 'A unified approach to mortality modelling using state-space framework: Characterisation, identification, estimation and forecasting', *Annals of Actuarial Science* **11**(2), 343–389.

Yaari, M. E. (1965), 'Uncertain lifetime, life insurance, and the theory of the consumer', *The Review of Economic Studies* **32**(2), 137–150.
